# A Case of Extensive Maxillary Medication‐Related Osteonecrosis of the Jaw With Wound Closure Using a Pedicled Buccal Fat Pad

**DOI:** 10.1155/crid/6687970

**Published:** 2026-03-02

**Authors:** Ryohei Iwasaki, Karen Gomi, Yusuke Kurosawa, Akinori Moroi, Kunio Yoshizawa, Koichiro Ueki

**Affiliations:** ^1^ Department of Oral and Maxillofacial Surgery, Division of Medicine, Interdisciplinary Graduate School, University of Yamanashi, Chuo, Yamanashi, Japan, yamanashi.ac.jp

## Abstract

Reports of extensive medication‐related osteonecrosis of the jaw (MRONJ) with pedicled buccal fat pad wound closure without tissue reconstruction are rare. We present the case of a 78‐year‐old man with prostate cancer and extensive MRONJ on the left side of the maxilla. The patient was treated with denosumab for the multiple bone marrow and lymph node metastases. Aggressive resection of the infected bone should be considered to prevent the spread of the infection if it extends into the maxillary sinus. In cases of extensive removal of decayed bone, jaw reconstruction is necessary to address the bone defect because it impairs the ability to eat, speak, and communicate. However, it was possible to close the wound and improve oral function by using a pedicled buccal fat pad for extensive MRONJ without maxillary reconstruction.

## 1. Introduction

Medication‐related osteonecrosis of the jaw (MRONJ) can be triggered by several antiresorptive and antiangiogenic medications, including bisphosphonates (BRONJ), denosumab (DRONJ), and other medications used to treat osteoporosis and metastatic bone cancer [1]. Henien et al. [2] reported that osteonecrosis of the jaw, which is not a lymphoproliferative disease, occurs in patients receiving long‐term methotrexate therapy without concomitant use of bone resorption inhibitors or angiogenesis inhibitors. In the present case, denosumab was administered for a long period, and it was thought that this drug may have been involved in the development of MRONJ. In recent years, reports of MRONJ in patients treated for cancer or osteoporosis have emerged [3]. MRONJ is caused by bone exposure resulting from treatment that is invasive to the jawbone at the site and so on. Mandibles have thicker cortical bone and sparser blood flow than the maxilla, which is why MRONJ occurs more frequently in the mandible. Therefore, MRONJ has been reported less frequently in the maxilla, and more extensive cases are rare [4].

When MRONJ was first named in 2003, conservative treatment was the preferred option, and aggressive surgical intervention was not recommended. However, in recent years, reports of successful surgical treatment have increased [5]. Nevertheless, there is no established method for determining the extent of bone removal and wound treatment. Even if a patient is cured through surgery, significant deterioration of oral function can be expected if extensive jaw resection is performed. Tissue reconstruction is required when extensive jaw resection is necessary. In this report, we present a case in which a wound was closed using buccal fat.

## 2. Case Presentation

**Figure 1:**
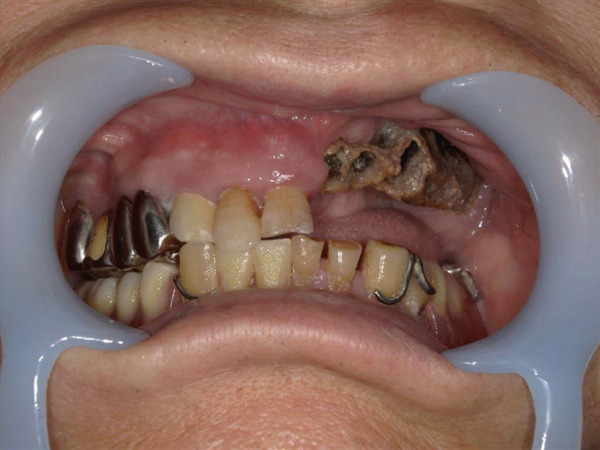
Intraoral photographs at initial examination.

**Figure 2:**
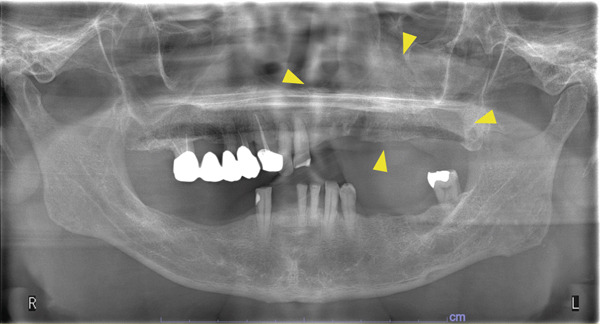
Panoramic image at initial examination.

**Figure 3:**
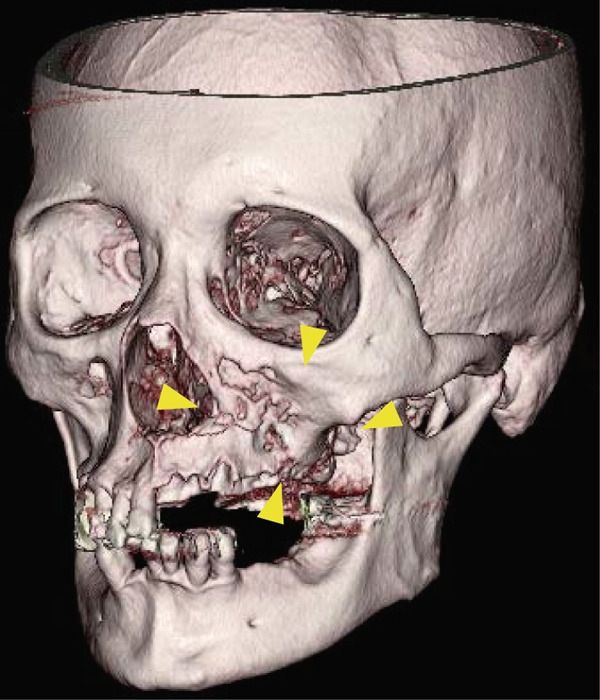
Preoperative 3DCT images.

**Figure 4:**
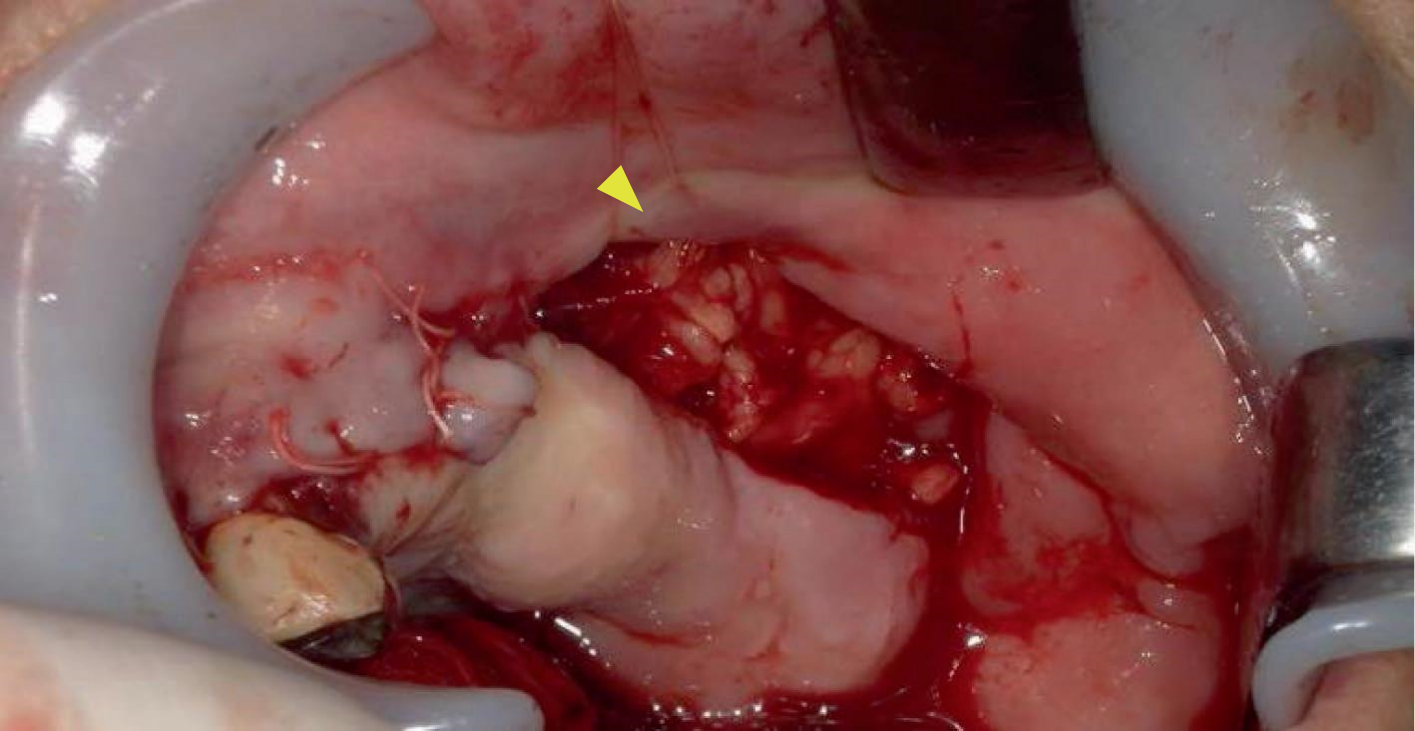
Intraoral images during the necrotic bone removal.

**Figure 5:**
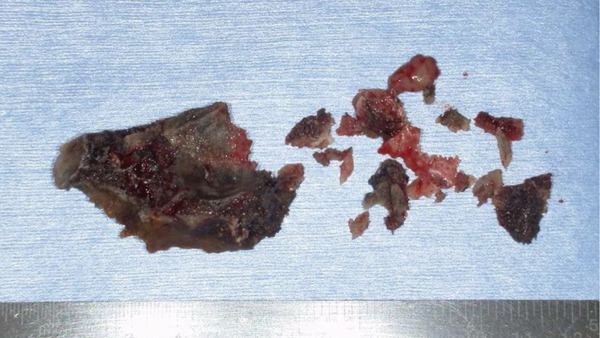
Surgically removed the necrotic bone.

A 78‐year‐old man presented to the Department of Oral and Maxillofacial Surgery, University of Yamanashi Hospital, complaining of bone exposure in the left maxilla. He had a history of bone metastasis from prostate cancer for more than 4 years and was receiving denosumab subcutaneous injection at 120 mg/1.7 ml. His medical history included diabetes mellitus, hypertension, angina pectoris, and dyslipidemia, and he was allergic to atenolol, cephem, and penicillin. The face was symmetrical, and drainage occurred from the exposed left maxilla. The local lymph nodes were normal, and there was no trismus. An intraoral examination revealed gingival swelling, erythema, and purulent discharge (Figure 1). Panoramic radiography revealed extensive necrotic bone fragments in the left maxilla (Figure 2). Computed tomography (CT) revealed bone sclerosis extending from the right maxillary lateral incisor region to the left maxillary premolar region, anterior nasal floor, and left maxillary palatal region, along with necrotic bone from the left maxillary anterior region to the molar region, indicating maxillary osteonecrosis and sinusitis (Figure 3). The administration and cleaning of the exposed bone with povidone‐iodine did not relieve the symptoms, and the patient underwent left maxillectomy under general anesthesia with drainage of the maxillary sinus. The buccal fat pad was bluntly dissected from the full‐layer valve, and the defect was covered with the left buccal fat pad and tied over with a Terramamycin ointment gauze (Figures 4 and 5).

**Figure 6:**
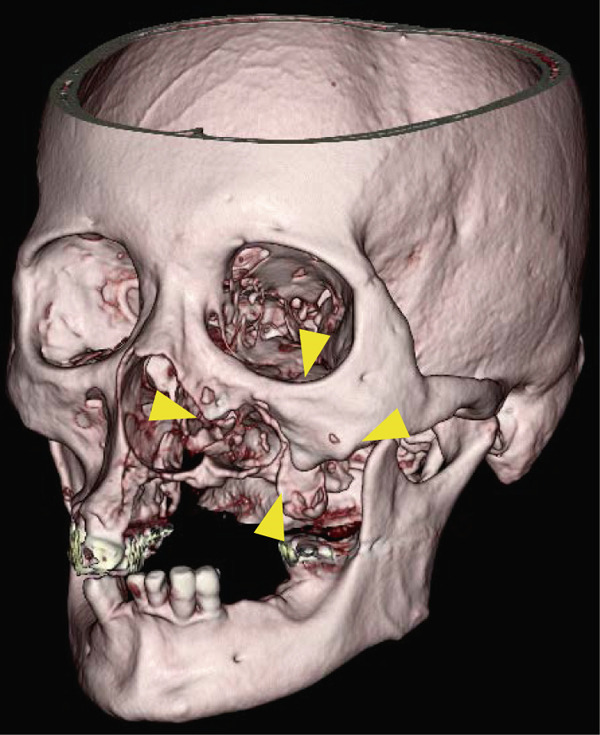
Postoperative 3DCT images.

**Figure 7:**
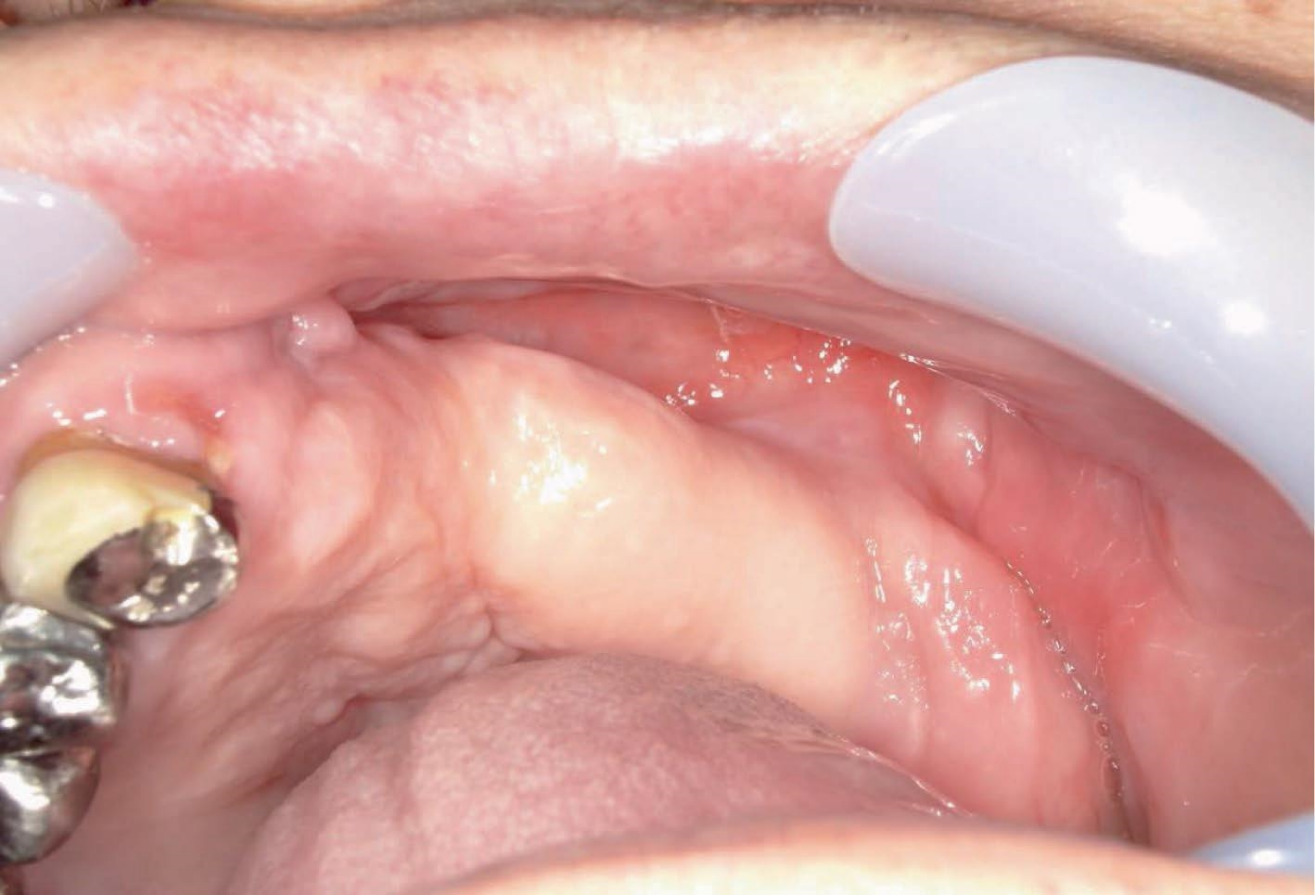
Intraoral image after the necrotic bone removal.

**Figure 8:**
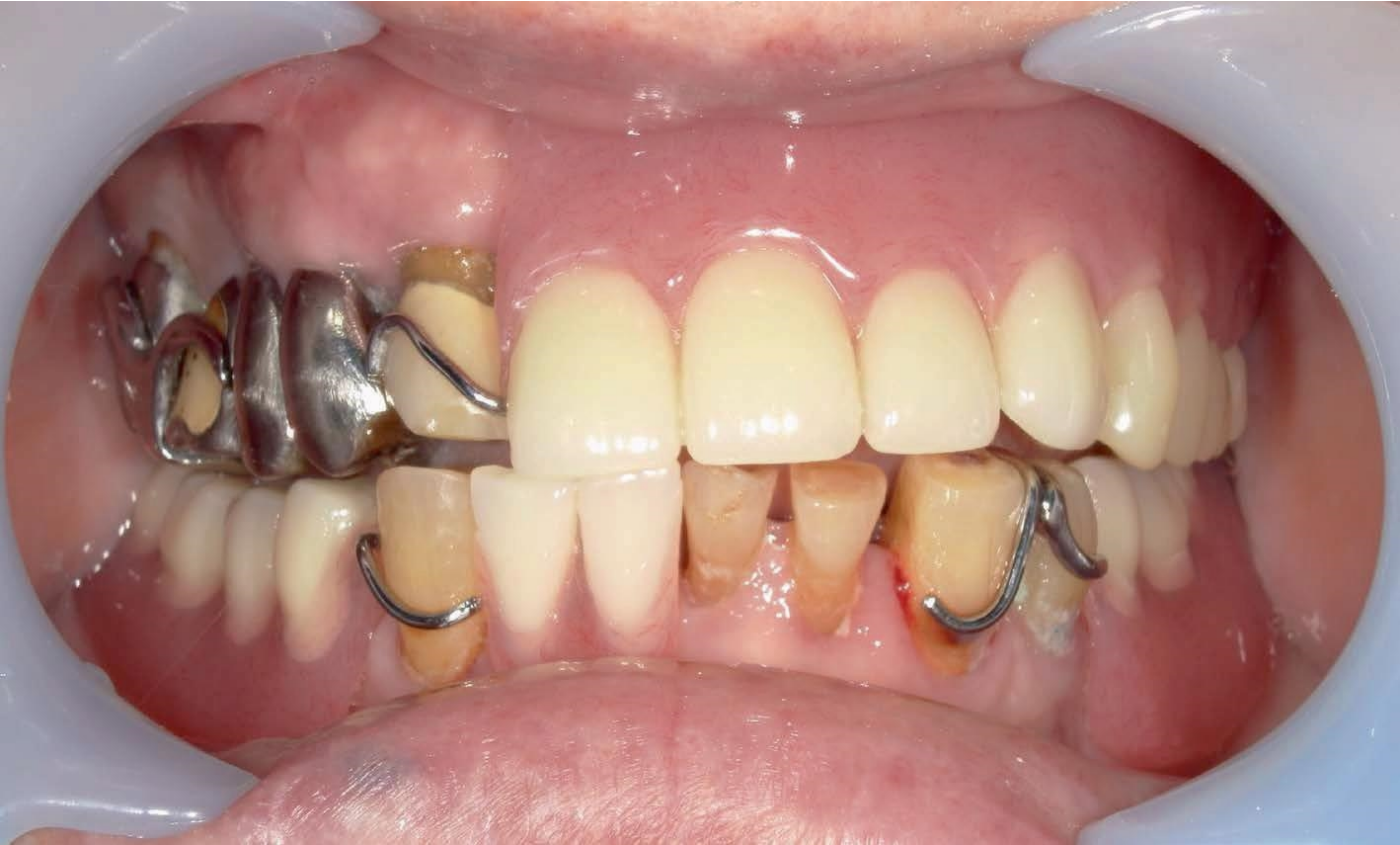
Maxilla denture for bone defects after removal of the necrotic bone.

The patient′s 10‐day postoperative course was uneventful, and he was discharged from the hospital. Subsequent CT revealed no residual necrotic bone (Figure 6). The wound epithelialized 1 month after surgery. After epithelialization, the prefabricated prosthesis was immediately fitted. 3 months after the surgery, no maxillary sinus fistulas were observed. The oral cavity was in good condition (Figure 7). There were no problems with the fit of the maxillary denture to the bone defect, and the patient had good occlusion (Figure 8).

## 3. Discussion

In 2003, Marx described the first case of osteonecrosis of the jaw after bisphosphonate treatment, which manifested as painful bone exposure of the jaw that was unresponsive to medical and surgical therapies [6]. He attributed it to the use of bisphosphonates such as pamidronate and zoledronate, prescribed to patients. The diagnosis of MRONJ is defined by the American Association of Oral and Maxillofacial Surgeons (AAOMS) [7], and our understanding of this disease has advanced significantly over the past decade. Antiresorptive drugs, including bisphosphonates and denosumab, are increasingly associated with this condition. Angiogenesis inhibitors, certain anticancer agents, tyrosine kinase inhibitors (TKIs), mammalian targets of rapamycin inhibitors, and immunotherapeutic agents have also been implicated in MRONJ [8]. The pathogenesis of MRONJ is not well understood [9]; however, the inhibition of bone resorption and alterations in bone remodeling are thought to play significant roles in the pathogenesis of MRONJ. The rapid rate of alveolar bone remodeling contributes to the high incidence of MRONJ in the jaw. The presence of inflammation of the periodontal ligament and the functional loading of the alveolar bone by dentition further increase the demand for bone remodeling in the alveolar bone, thus heightening dependence on osteoclast‐mediated bone resorption and remodeling. Periodontal infection, tooth extraction, and ill‐fitting dentures can also precipitate MRONJ [10].

In the present case, the patient had been using denosumab for more than 4 years, which suppressed bone metabolism. Risk factors such as periodontal infection and tooth extraction may have further increased the need for bone remodeling, leading to the development of MRONJ.

Various medical and surgical treatments have been attempted to manage MRONJ. Traditionally, the management of MRONJ has been aimed at controlling infection and preventing further progression of necrosis [11]. In the early stages, such as when there is no clinical evidence of necrotic bone but nonspecific clinical findings, radiographic changes, and symptoms (Stage 0), or when there is exposed and necrotic bone or fistulae that probe to the bone in asymptomatic patients with no evidence of infection (Stage 1), conservative treatment is most advocated. Various local and systemic antimicrobial agents, such as pentoxifylline, vitamin E, hyperbaric oxygen therapy, and teriparatide, have been used with inconsistent results [12]. Surgical treatment is often reserved for patients with advanced stages that are refractory to medical therapy. Excision, debridement, and coverage of surgical defects using vascular flaps are the most commonly performed surgical procedures. Complete coverage of the exposed bone is essential to prevent disease recurrence and progression [13].

Preoperative CT images revealed that the inflammation in this case involved the mucosa of the left maxillary sinus floor and extended from the left maxillary dentition across the center to the right maxillary canine. Compared with previous cases (Table 1), the degree of bone collapse was relatively extensive, and conservative treatment was judged to be difficult owing to the expected expansion of the lesion in the future; thus, the decision was made to proceed with surgical treatment. In the present case, because the gingival mucosa was preserved as much as possible, we decided to use a buccal fat body with a pedicle for wound closure.

**Table 1:** Cases of decomposition osteotomy of the maxilla.

	**Gender**	**Age(years)**	**Chemotherapeutic drug**	**Defect size(mm)**	**Treatment**
André Ferreira Leite (2006) [4]	Male	82	Zolendronic acid	34 × 14	Primary closure with intranasal
Jan Rustemeyer (2014) [14]	Male	44	Methamphetamine	Undocumented	Primary closure with intranasal
Yoshinari Myoken (2022) [15]	Female	64	Alendronic acid	30 × 50	Buccal fat pad flap
Andreas Sakkas (2021) [16]	Female	45	Tocilizumab	Undocumented	Primary closure with intranasal
Panagiotis Koulocheris (2008) [17]	Male	49	Zolendronic acid	48 × 20	Primary closure with intranasal
Luiz Fernando Mathias Duart (2015) [18]	Female	58	Zolendronic acid	Undocumented	Buccal fat pad flap

Because recent studies have shown that subcutaneous adipose tissue differentiates into multiple lineages and expresses multiple growth factors, a buccal fat pad (BFP) was performed [19]. Mucosal healing is impaired in MRONJ. This is predominantly because of the soft tissue effects of these drugs. These medications have also been associated with decreased proliferation and induction of apoptosis in keratinocytes and fibroblasts, resulting in impaired healing and wound differentiation. Furthermore, the poor vascularity of the mucosal flap can also be attributed to poor wound healing. Therefore, the chances of wound dehiscence and impaired wound healing are relatively high when only a mucosal flap is used for coverage.

There are a few reports of extensive MRONJ of the maxilla, most of which involved reconstructive surgeries (Table 1). Some cases ranged in size from 31 × 14 mm to 30 × 50 mm. In the present case, the rotator bone was 63 × 24 mm in size, and there were no cases of MRONJs in this size range that were closed using only layer BFP without tissue reconstruction. In this case, a maxillary denture was created for the bone defect to restore oral function. After surgical treatment, occlusal reconstruction is essential for improving the patient′s quality of life. Functional reconstruction with normal dentures is often difficult with occlusal reconstruction, and continuous follow‐up is essential.

In conclusion, determining whether tissue reconstruction is necessary for extensive MRONJ is difficult. However, buccal fat bodies may be an option for postoperative occlusal recovery.

## Funding

No funding was received for this manuscript.

## Conflicts of Interest

The authors declare no conflicts of interest.

## Data Availability

The data that support the findings of this study are available on request from the corresponding author. The data are not publicly available due to privacy or ethical restrictions.
